# Hydrogel Heart Model with Temperature Memory Properties for Surgical Simulation

**DOI:** 10.3390/s19051102

**Published:** 2019-03-04

**Authors:** Hisataka Maruyama, Yuki Yokota, Keisuke Hosono, Fumihito Arai

**Affiliations:** Department of Micro-Nano Mechanical Science and Engineering, Nagoya University, Furo-cho, Chikusa-ku, Nagoya, Aichi, 464-8601, Japan; hisataka@mech.nagoya-u.ac.jp (H.M.); hosono@biorobotics.mech.nagoya-u.ac.jp (K.H.); arai@mech.nagoya-u.ac.jp (F.A.)

**Keywords:** hydrogel model, temperature measurement, surgical simulator, heart model

## Abstract

The continual development of surgical technology has led to a demand for surgical simulators for evaluating and improving the surgical technique of surgeons. To meet these needs, simulators must incorporate a sensing function into the organ model for evaluating the surgical techniques. However, it is difficult to incorporate a temperature sensor into the conventional cardiac training model. In this study, we propose a heart model for surgical training of cardiac catheter ablation made from hydrogel, which has temperature memory properties. The heart model consists of a photo-crosslinkable hydrogel mixed with an irreversible temperature indicator that exhibits a color change from magenta to colorless at 55 °C. The Young’s modulus, electrical resistivity, thermal conductivity, and specific heat capacity of the hydrogel material were evaluated and compared with those of human heart. Furthermore, temperature calibration based on the color of the hydrogel material confirmed that the temperature measurement accuracy of the material is ±0.18 °C (at 56 °C). A heart model for catheter ablation was fabricated using the hydrogel material and a molding method, and the color change due to temperature change was evaluated.

## 1. Introduction

Progress in medical technology and surgical instruments must be supported by the high surgical skill of surgeons. For this reason, there is a growing need for an artificial organ model and surgical simulator for developing surgical skills [1,2,3]. Conventionally, animal models with similar structure and characteristics to human organs, VR simulators that use virtual reality, and artificial organ models made of synthetic materials that mimic the structure and characteristics of human organs have been used. Animal models have limitations in their similarities—both structural and in terms of physical properties—to human organs [4,5,6,7]. There are also ethical constraints in the use of animal models. While VR simulators can simulate the surgical process without physical models, it is not possible to use specific surgical instruments and interfaces, and registration of new surgical instruments takes time and has a considerable cost [8]. In contrast, artificial organ models have several advantages over other surgical simulators, such as no ethical issues and the possibility of simulating structures and properties similar to those of the target organ. The progress of artificial organ models has been marked and numerous surgical simulators with artificial organ models have been developed and commercialized [9,10,11,12]. However, most artificial models do not have sensors for evaluating the skill of the surgeon or the properties of surgical instruments. The integration of measurement functions into artificial organ models without sacrificing the similarity of the structures and properties to those of the target is a promising approach to improving the quality of surgical training by giving quantitative evaluation of the skill of the surgeon or the properties of the surgical instruments [13].

Catheter ablation is a procedure for which a surgical simulator with measurement capability is required. In catheter ablation, an electrode is inserted into the target heart blood vessel and ablation energy is applied to the myocardial tissue—the cause of the arrhythmia—via the catheter to cauterize the tissue [9]. The target temperature of the heated area is 55 °C and treatment by high-frequency ionization to the atrioventricular junction is mainstream [14,15,16]. Three-dimensional mapping techniques of perfusion catheters and the development of catheters that perfuse the tip of the electrode with cooling water have contributed to improving the safety of catheter ablation. [11,12]. However, catheter ablation at the atrium remains difficult and has a high risk of complications. Particularly serious complications are heart tamponade and esophageal fistula [17]. Cardiac tamponade is the most frequently occurring complication and is caused by atrial puncture with an electrode catheter and excessive cauterization of myocardial tissue. Esophageal fistula has the highest fatality rate and involves the formation of through holes between the left atrium and the esophagus due to excessive cauterization of the posterior wall of the left atrium [18]. Surgical training for catheter ablation using an artificial heart model with a temperature measurement function is, therefore, important for avoiding these risks.

Some approaches to integrating temperature measurement functionality into the artificial heart model are introducing thermocouples, temperature measurement of the model surface using a thermal camera, and integration of temperature sensitive dye. Thermocouples can measure temperature with high sensitivity and high accuracy and thermal cameras can measure the temperature distribution of the model surface in real time. However, these measurement methods have several limitations for temperature measurement in artificial heart models. The integration of thermocouples that are made of metal into the artificial heart model requires cables for measurement, and integration of high thermally-conductive material may change the mechanical, electrical, and thermal properties of the model. The thermal camera cannot measure the temperature of the artificial heart model if it is suspended in water, since the camera measures the temperature at the water surface. In addition, a camera cannot measure the maximum temperature reached inside the artificial heart model.

Our group developed an artificial renal artery with temperature indication. The silicone artery model was impregnated with irreversible temperature-indicating dye [19]. The maximum temperature reached by the artery model was measured by color analysis of the material with image processing. The Young’s modulus of the artery model was reproduced similar to the human renal artery. However, the model was made of silicon, and the electrical properties of the model were not similar to those of the human artery. For application in catheter ablation simulation, the material of the artificial organ model should reproduce the electrical resistivity of the target human organ.

In this study, we propose a hydrogel heart model with temperature memory properties for catheter ablation training. The hydrogel heart model mainly consists of a photo-crosslinkable hydrogel that reproduces the Young’s modulus and the electrical resistivity of human heart for catheter ablation. The hydrogel heart model is designed to detect areas where the temperature reaches 55 °C or higher through the color change of the hydrogel. Irreversible temperature-indicating dye is impregnated into the hydrogel material to evaluate the distribution of temperature reached in the hydrogel heart model after training. An irreversible temperature-indicating dye, which changes from magenta to colorless around 55 °C, was used. In addition, it is also possible to observe the areas where the temperature reaches 55 °C or higher during training. The Young’s modulus, electrical resistivity, thermal conductivity, and specific heat capacity of the hydrogel material were measured and compared with those of human heart. The color of the hydrogel material was calibrated with temperature using optical imaging. Finally, we fabricated an artificial heart model of the left atrium with temperature memory functionality using a molding and photo-polymerization method.

## 2. Materials and Methods

### 2.1. Hydrogel Heart Model with Temperature Memory Properties

**Figure 1:**
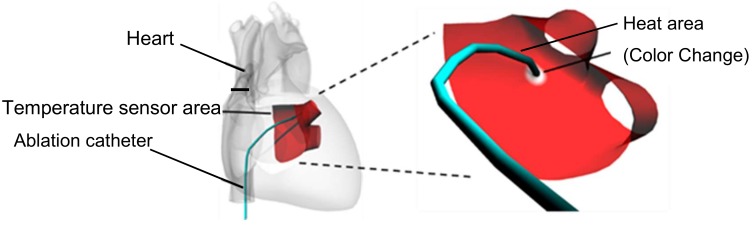
Schematic diagram of the heart model with temperature sensing.

Figure 1 shows a schematic diagram of the hydrogel heart model for recording the distribution of maximum temperature reached. The hydrogel heart model has a junction with the pulmonary vein of the left atrium where ablation is conducted in the treatment of arrhythmia. In arrhythmia treatment, the ablation catheter is pressed against the target area to cauterize and block the stray current flow from the pulmonary vein to the left atrium by heating with a high-frequency current. The target area is heated to 55 °C or higher to cauterize the tissue. The requirements of a heart model for simulating catheter ablation are as follows.

1. Reproduction of the Young’s modulus of the human heart;
2. Reproduction of the electrical resistivity of the human heart;
3. Reproduction of the thermal conductivity and specific heat capacity of the human heart;
4. Detection of the areas at which the temperature exceeds 55 °C.

Conventionally, silicone, urethane, and polyvinyl alcohol have been used as materials for artificial heart models. These materials are intended to simulate mechanical properties, such as the shape and Young’s modulus of the heart model, and are not subject to the simulation of electrical resistivity or thermal characteristics. Silicone and urethane are insulators, so they cannot be used for surgical training of high-frequency electrical heating.

In this study, hydrogel material was chosen for the heart model to reproduce the Young’s modulus, electrical resistivity, and thermal properties of heart tissue. The Young’s modulus of the hydrogel heart model was adjusted by altering the concentration of the hydrogel. The electrical resistivity of the hydrogel heart model was adjusted by altering the electrolyte concentration in the hydrogel. The thermal properties were determined from the constitution of the hydrogel material. An irreversible temperature indicator was impregnated into the hydrogel material for detection of the areas of the hydrogel heart model that were heated over 55 °C. A water-soluble polyurethane, with a photo-crosslinkable resin, was used as the main constituent of the hydrogel material. The hydrogel heart model was fabricated using a molding method and photo-polymerization. The color of the hydrogel material changed from magenta to colorless around 55 °C. After the color had changed, it did not recover to magenta. Therefore, the areas where the temperature increased above 55 °C were permanently indicated by the color change of the hydrogel, allowing for visual inspection. Quantitative measurement of the hydrogel heart model was conducted by cutting out the target areas of the model with a specific thickness, followed by analysis of microscopy images using color information.

### 2.2. Materials for Hydrogel Heart Model Preparation

The materials used to prepare the hydrogel heart model were water-based polyurethane (PU-W2A, Shin-Nakamura Chemical Co., Ltd., Wakayama, Japan), 20% polyvinyl alcohol (PVA 424H, KURARAY Co., Ltd., Tokyo, Japan) in aqueous solution, pure water, sodium chloride (Wako Pure Chemical Industries, Ltd.), dimethyl sulfoxide (DMSO, Wako Pure Chemical Corporation, Osaka, Japan), photo-initiator (Lithium phenyl (2,4,6-trimethyl-benzoyl) phosphate, Tokyo Chemical Industry Co., Ltd., Tokyo, Japan), and an irreversible temperature indicator solution (METAMO COLOR, PILOT CORPORATION, THE PILOT INK COMPANY, LIMITED, Aichi, Japan). The electrical resistivity was adjusted using the concentration of sodium chloride. The Young’s modulus of the heart model was adjusted using the concentration of polyurethane, polyvinyl alcohol, and photo-initiator. The color of the temperature indicator was magenta at room temperature and colorless at temperatures above 55 °C. This color change did not recover when the temperature recovers to room temperature. Table 1 shows the composition of each material used for the hydrogel heart model.

## 3. Evaluation of Hydrogel Material

### 3.1. Young’s Modulus of the Hydrogel Material

The Young’s modulus of the hydrogel material was evaluated by tensile tests using Equation (1).

(1) $$E=\frac{\sigma }{\varepsilon }=\frac{F}{A}\cdot \frac{L_{0}}{dL}$$

where *E* is Young’s modulus, *σ* is stress, and *ε* is strain, *F* is the force input to the specimen, *A* is the cross-sectional area of the specimen, *L_0_* is the initial length of the specimen, and *dL* is the displacement of the specimen. A specimen of the hydrogel material with a thickness of 2 mm was fabricated using a dumbbell cutter (SDMP-1000, DUMBBELL Co., Ltd, Saitama, Japan). The tensile test was carried out using a tensile tester EZ-LX (Shimadzu corporation, Kyoto, Japan), and the Young’s modulus was calculated using the result of the tensile test and the cross-sectional area and strain of the specimen.

**Figure 2:**
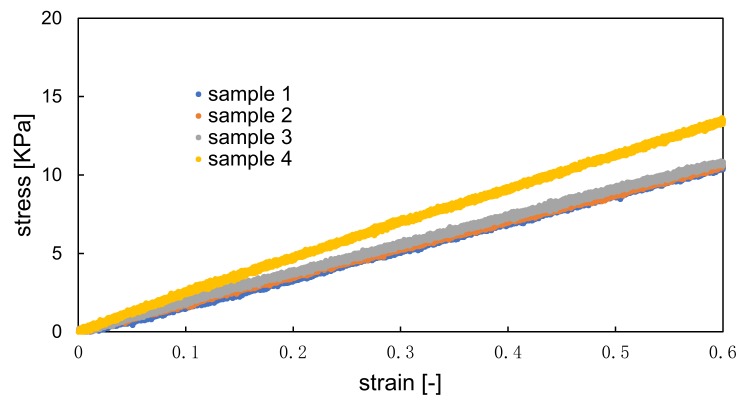
Tensile test of hydrogel material.

Figure 2 shows the tensile test of the hydrogel material. The Young’s Modulus of the hydrogel material was calculated using the value of 10 % strain condition of stress-strain curve in Figure 2 and Equation (1). From the results of the tensile test using four specimens made of the hydrogel material, shown in Table 1, the Young’s modulus was measured to be 20.0 ± 3.3 kPa, which is similar to the value of human heart from the literature shown in Table 2 [20,21,22]. 

### 3.2. Electrical Resistivity of the Hydrogel Material

**Figure 3:**
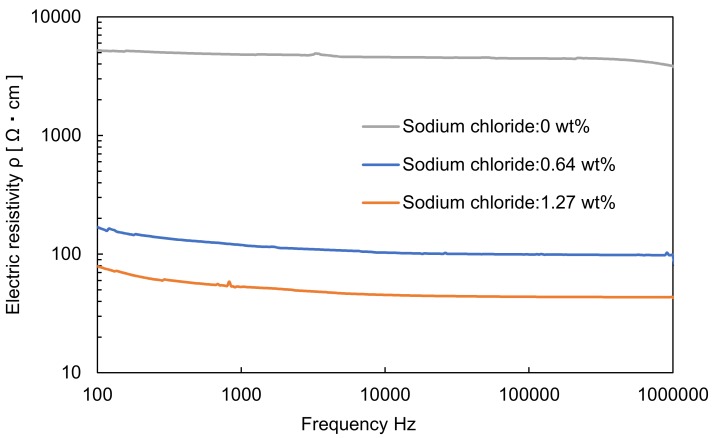
Hydrogel electrical resistivity for different sodium chloride concentrations.

The electrical resistivity of the hydrogel material was evaluated using Equation (2).

(2) $$R=\rho \frac{l}{M}$$

where *R* is the electrical resistance of the hydrogel material, *ρ* is the electrical resistivity of the hydrogel material, *l* is the length of the hydrogel material, and *M* is the cross-sectional area of the hydrogel material. An Impedance Analyzer ZA 5403 (NF corporation, Kanagawa, Japan) was used to measure the electrical resistance. The size of the specimen was 2 mm in width and 2 mm in thickness. The length was measured for each specimen. The frequency for measuring the electrical resistance was 100 Hz to 10 MHz. The electrical resistivity was measured for hydrogel materials prepared with different concentrations of sodium chloride (0 wt%, 0.64 wt%, and 1.27 wt%) to evaluate the effect of sodium chloride concentration on electrical resistivity (Figure 3). The hydrogel material containing 0.64 wt% sodium chloride is the same constitute shown in Table 1. The electrical resistivity of the human heart in the literature is 132 Ω·cm at 1 MHz, as shown in Table 2 [20,21,22]. The average value of the electrical resistivity at 1 MHz was 3.85 × 10^2^ Ω∙cm in 0 wt% sodium chloride. The average value of the electrical resistivity at 1 MHz decreased to 43.5 Ω∙cm in 1.27 wt% sodium chloride. These results show that the electrical resistivity can be controlled by adjusting the concentration of sodium chloride. In this study, the concentration of sodium chloride used was 0.64 wt% and the average electrical resistivity at 1 MHz was 98.1 Ω∙cm. This value is similar to the electrical resistivity of human heart tissue [22,23,24].

### 3.3. Thermal Properties of the Hydrogel Material

The thermal properties of the hydrogel material were evaluated by determining the thermal conductivity and specific heat capacity. To measure thermal conductivity, the laser flash, steady state, or heat ray methods can be applied. In this study, the heat ray method, which can measure within a short time period, was used to reduce the influence of drying of the hydrogel material. Measurement of the thermal conductivity was carried out using a thermal conductivity measuring device TCi (Rigaku Corporation, Tokyo, Japan), and the size of the hydrogel specimen was 33 mm in diameter and 1 cm in height. The measured thermal conductivity was 1.33 W/m∙k, as shown in Table 2 [20,21,22]. This value was higher than the thermal conductivity of the human heart. 

**Figure 4:**
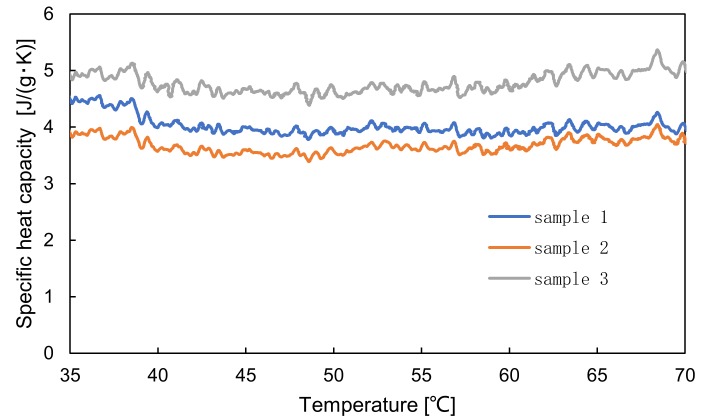
Specific heat capacity of the hydrogel material.

The specific heat capacity of the hydrogel material was measured using heat flux differential scanning calorimetry. The temperature difference between the test material and a reference substance was measured while varying the temperature of a sample composed of the test material and reference substance. The specific heat capacity of the sample was calculated from the measured data. A DSC-60A (Shimadzu corporation, Kyoto, Japan) was used for measurement. To prevent the effect of drying, the sample was introduced into a chamber cell and sealed. The range of temperature measurement was 30–70 °C and the heating rate was 1 °C/min. Figure 4 shows experimental data for 3 specimens made of the hydrogel materials shown in Table 1. The average value of specific heat capacity at 56 °C was 4.1 J/(g·K), and this value was similar to that of human heart, as shown in Table 2 [20,21,22].

### 3.4. Calibration of Hydrogel Material Color with Temperature

**Figure 5:**
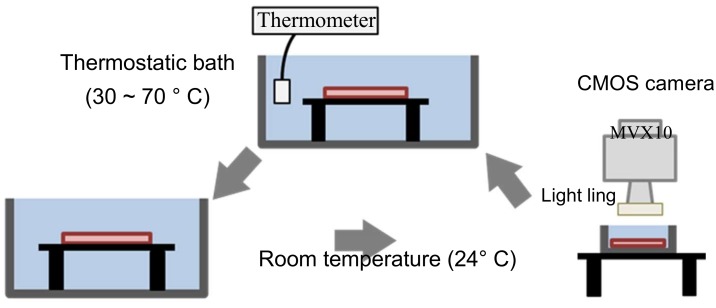
Schematic diagram of the calibration process.

The area of the hydrogel heart model heated over 55 °C was determined from the color of the hydrogel. Calibration of hydrogel material color with temperature reached was performed using the experimental setup shown in Figure 5. Images of the hydrogel material were acquired with a CMOS color camera (GS3-U3-23S6C-C, FLIR Systems, Inc., Wilsonville, OR, USA), using an objective lens (MV PLAPO 1X, Olympus corporation, Tokyo, Japan) with a magnification of 1 in a stereomicroscope (MVX 10, Olympus Corporation, Tokyo, Japan). LED lighting (LED-R72, ARMSSYSTEM corporation, Tokyo, Japan) was used as the light source. To eliminate the influence of the thickness of the specimen, the thickness was fixed at 2 mm. A water bath type thermostatic chamber (TR-3A, As One corporation, Osaka, Japan) was used for heating the hydrogel material, and the water temperature was measured with a thermocouple (CTH-1365, CUSTOM corporation, Tokyo, Japan). The temperature of the water in the thermal bath was controlled for heating the hydrogel material. The hydrogel material was sink in the thermal bath, and kept for 5 min to make the temperature of the hydrogel material the same as that of the water in the thermal bath.

The acquired image is represented in the RGB color space. In the RGB color space, brightness information is included in the R, G, and B values of each pixel. Therefore, measurement using the RGB color space is influenced by fluctuations of the illumination light. To eliminate this effect, the RBG color space was converted to the YCrCb color space using Equation (3) to separate the brightness information and color information in each pixel.

(3) $$\left[\begin{array}{c} Y\\ Cr\\ Cb \end{array}\right]=\left[\begin{array}{ccc} 0.299 & 0.587 & 0.114\\ 0.500 & -0.419 & -0.081\\ -0.169 & -0.331 & 0.500 \end{array}\right]\left[\begin{array}{c} R\\ G\\ B \end{array}\right]$$

*R* is red, *G* is green, *B* is blue, *Y* is brightness, *Cr* is red color difference, and *Cb* is blue color difference. The temperature calibration process for the hydrogel material is shown in Figure 5.

1. The hydrogel material was immersed in the thermostatic chamber at 30 °C for 5 min;
2. The hydrogel material was removed from the chamber and put in water at 24 °C for 5 min;
3. The hydrogel material was moved from the water to the microscope and the image was acquired;
4. The above process was repeated from 30–70 °C. In the range 55–60 °C, temperature was changed in 0.5 °C intervals.

**Figure 6:**
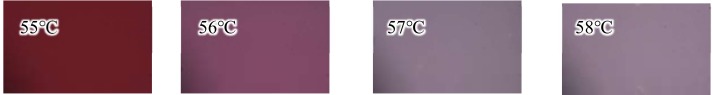
Color of the hydrogel material at each temperature.

**Figure 7:**
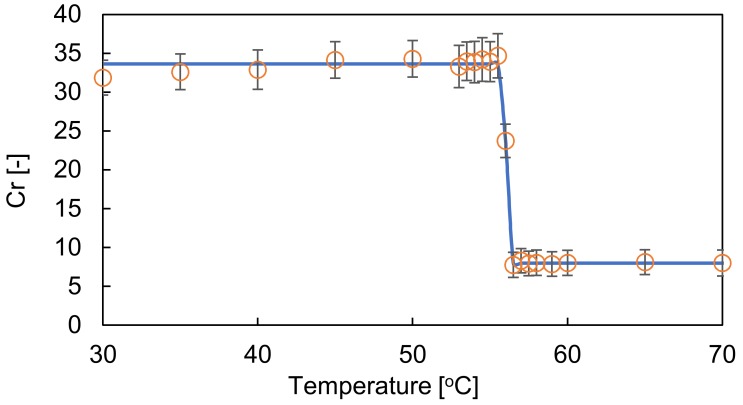
Calibration of color with temperature.

Figure 6 shows images of the hydrogel material in the temperature range 55–58 °C. The color of the hydrogel material changed between 55.5 and 56.5 °C. Figure 7 shows the results of the calibration of Cr with temperature from 30 to 70 °C. The calibration results were fitted using the sigmoid function shown in Equation (4). Table 3 shows parameters as a result of calibration to the sigmoid function.

(4) $$T_{\max}=c+\frac{1}{b}\ln\frac{Cr-d}{a}$$

where *T*_max_ is the measured temperature of the water in the thermal bath, and *a*, *b*, *c*, and *d* are parameters of the sigmoid function. The calibration result confirmed that the temperature reached by the hydrogel material can be measured with high accuracy ± 0.18 °C at 56 °C.

## 4. Fabrication of Hydrogel Heart Model with Temperature Memory Functionality

As a hydrogel heart model, we fabricated part of the left atrium with the pulmonary vein. This area is a target region in catheter ablation for treatment of arrhythmia. A molding method and photo-polymerization were used for fabrication. Polydimethylsiloxane (PDMS), which is a transparent silicone elastomer, was used as a mold for the heart model. The photo-crosslinkable hydrogel material was polymerized using ultraviolet (UV) illumination as PDMS transmits UV light. In addition, an oxygen inhibition layer on the inner wall surface of the PDMS was formed, since PDMS has high oxygen permeability. This oxygen inhibition layer inhibits the photo-polymerization, and prevents polymerized hydrogel adhering to the PDMS mold. Therefore, the fabricated hydrogel heart model can be easily removed from the PDMS mold.

**Figure 8:**
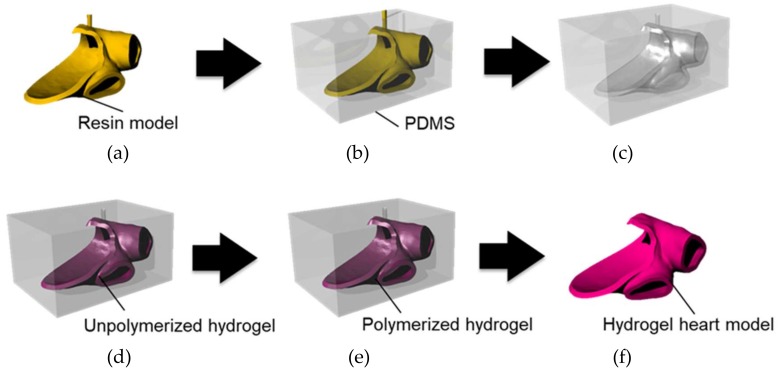
Hydrogel heart model fabrication process. (**a**) Fabrication of a resin heart model, (**b**) introduction of the resin model into PDMS, (**c**) removal of the resin model from the polymerized PDMS, (**d**) introduction of uncured hydrogel material, (**e**) photo-polymerization of the hydrogel material by UV irradiation, and (**f**) extraction of the cured hydrogel heart model from the PDMS mold.

Figure 8 shows the fabrication process for the hydrogel heart model. The 3D data of the cardiac model was generated from CT scan data of the heart.

1. A resin model of a heart model was fabricated from 3D data using a commercial 3D printer.
2. The resin model was introduced into uncured PDMS.
3. After curing the PDMS, the resin model was removed to make a hollow PDMS mold.
4. Unpolymerized hydrogel material was introduced into the PDMS mold.
5. The hydrogel material is irradiated with UV (375–405 nm) light from the periphery of the PDMS mold for photo-polymerization of the hydrogel.
6. The polymerized hydrogel heart model was removed from the PDMS mold.

**Figure 9:**
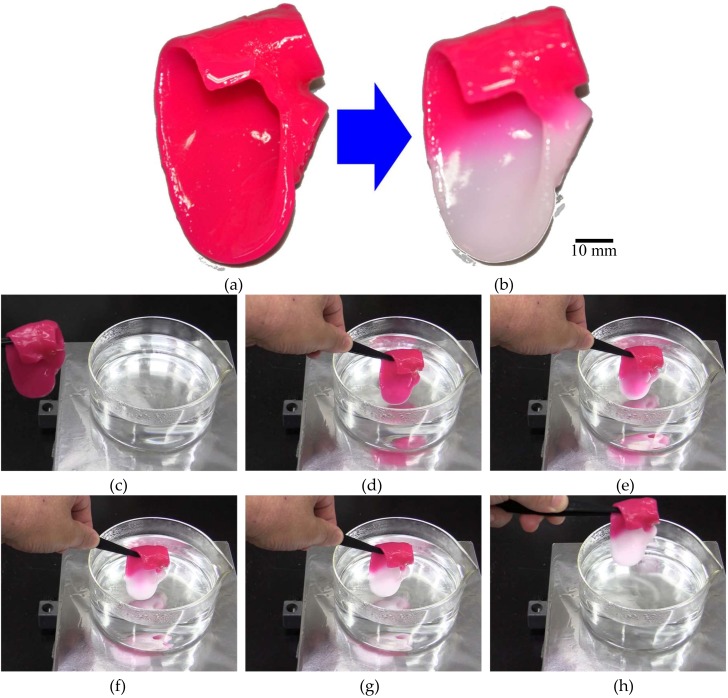
Photographs of the fabricated hydrogel heart model. (**a**) Before heating, and (**b**) after heating the lower part of the model at 60 °C. (**c**) Before immersing the hydrogel heart model. (**d**) Immersing the hydrogel heart model, (**e**) after 10 s, (**f**) after 20 s, (**g**) after 30 s, and (**h**) picking up the hydrogel heart model.

A DLP type 3D printer (M3DS-SA5, MITS Electronics corporation, Tokyo, Japan) was used to fabricate the resin heart model. UV Curing Chamber (XYZ printing incorporated, Tokyo, Japan) was used for UV illumination. The wavelength of the UV LED was 375–405 nm. Power of UV LED was 180 mW at 400 nm. Emission exposure time was 30 s. The fabricated hydrogel heart model is shown in Figure 9a. When the lower half of the hydrogel heart model was immersed in hot water at 60 °C for 30 s, the immersed area changed color from magenta to colorless, as shown in Figure 9b. After immersing the hydrogel heart model to hot water, the color of the hydrogel heart model changed from magenta to colorless gradually. The color of the immersed area of the hydrogel heart model completely changed within 30 s. This discoloration was maintained when the temperature was subsequently reduced. These findings confirmed the effectiveness of the hydrogel heart model with temperature memory functionality.

## 5. Discussion

In this study, we developed a hydrogel heart model that reproduces the Young’s modulus, electrical resistivity, and thermal characteristics of human heart tissue for surgical training of catheter ablation. As shown in Table 3, we succeeded in reproducing the characteristics of human heart, such as Young’s modulus, electrical resistivity, and specific heat capacity. However, the thermal conductivity of the hydrogel material was three times higher than that of human heart tissue. Since the thermal conductivity of PVA is ~0.3 W/mK, that of the water-soluble polyurethane is 0.2 W/mK, and that of water is 0.58 W/mK, the thermal conductivity of the temperature indicator is thought to be much higher than that of human tissue [23,24]. The thermal conductivity of the hydrogel should, therefore, be reduced to more closely simulate the thermal conductivity of human heart [19]. One solution is to introduce other low thermal conductivity materials while considering their effect on the other properties, such as Young’s modulus, electrical resistivity, and specific heat capacity. Adjusting the thermal conductivity of the hydrogel material is an aim of the future work. We adjusted the physical parameter of the hydrogel model, as shown in Table 1, by trial and error. When we changed the constitute of the hydrogel, we faced the coupling problem of several parameters of the hydrogel material. For example, increase of the concentration of water-soluble PU resulted in increase of Young’s modulus and decrease of thermal conductivity of the model, since thermal conductivity of water-soluble PU is lower than that of the hydrogel material. Construction of a design theory considering such coupling problems is a future task.

In this work, the areas of the hydrogel heart model that reached the target temperature were indicated by a permanent color change of the hydrogel owing to the inclusion of an irreversible temperature indicator that changes color from magenta to colorless at temperatures over 55 °C. The accuracy of the temperature measurement in the discolored area was ±0.18 °C (at 56 °C), which enabled highly accurate analysis of the areas of the hydrogel that exceeded 55 °C. In addition, the distribution of the areas inside the model heated over 55 °C could be measured by taking sections. This hydrogel model with temperature indication could be applied to different simulations by changing the temperature indicator. For measurement over a wide temperature range, indication could be achieved by mixing different indicators [19]. In addition, a reversible temperature indicator could be used for real-time monitoring of temperature variation. As described above, various applications could be realized by changing the temperature indicator for the hydrogel material according to the application.

## 6. Conclusions

In this study, we proposed a hydrogel heart model, which has temperature memory properties, for surgical training of cardiac catheter ablation. The model reproduces the physical, electrical, and thermal characteristics of heart tissue, and the areas of the model heated over 55 °C were recorded. A tensile test showed that the Young’s modulus of the hydrogel material was similar to that of human heart tissue. For high frequency heating with an ablation catheter, the electrical resistivity was adjusted to a value close to that of the human heart using a hydrophilic photo-crosslinkable hydrogel and electrolyte. Thermal conductivity and specific heat capacity were evaluated to establish the thermal properties of the hydrogel material. The specific heat capacity was similar to that of human heart. However, the thermal conductivity was higher than that of human heart tissue and adjustment of the thermal conductivity will form part of our future work. The color of the hydrogel material changed from magenta to colorless at temperatures over 55 °C—the target temperature of catheter ablation—to give temperature memory properties. The color change of the hydrogel material was introduced using an irreversible temperature indicator. The evaluation of the area of the hydrogel model heated over 55 °C was performed by visual observation and image analysis. The color of the hydrogel material was calibrated with the temperature reached by image analysis using the YCrCb color space, and successfully measured an accuracy at 56 °C of ± 0.18 °C. The hydrogel heart model was fabricated using a molding method and photo-polymerization based on CT data of the left atrium of a patient. Since photo-polymerization was achieved by irradiating with UV light for 1 min, it is possible to fabricate a heart model in a short time. Color change due to heating of the hydrogel heart model was demonstrated.

In future work we will adjust the thermal conductivity of the model to that of the human heart and simulate catheter ablation with high-frequency current to confirm the effectiveness of our hydrogel model as a surgical simulator. This surgical model with sensing functionality will make a significant contribution to the medical field.

## Figures and Tables

**Table 1:** Composition of the hydrogel material.

Thermo-Sensitive Dye	Polyurethane	20%PVA	DI Water	NaCl	DMSO	Photo-Initiator
10 g	20 g	20 g	150 g	1.35 g	10 g	200 mg

**Table 2:** Comparison of the properties of the hydrogel model and human heart.

Parameter	Human Heart (Literature)	Heart Model
Young’s modulus [kPa]	20.3 [20]	20.0
Electric resistivity [Ω·cm]	132 [21]	98.1
Thermal conductivity [W/m·K]	0.47 [22]	1.33
Specific heat capacity [J/g·K]	3.55 [22]	4.0

**Table 3:** Parameters of sigmoid function.

a	b	c	d
20.0	7.98	25.6	56.0
